# Naked mole-rat transcriptome signatures of socially suppressed sexual maturation and links of reproduction to aging

**DOI:** 10.1186/s12915-018-0546-z

**Published:** 2018-08-02

**Authors:** Martin Bens, Karol Szafranski, Susanne Holtze, Arne Sahm, Marco Groth, Hans A. Kestler, Thomas B. Hildebrandt, Matthias Platzer

**Affiliations:** 1 Leibniz Institute on Aging - Fritz Lipmann Institute, Beutenberg Str. 11, 07745 Jena, Germany; 2 Leibniz Institute for Zoo and Wildlife Research, Alfred-Kowalke-Straße 17, 10315 Berlin, Germany; 3 Institute of Medical Systems Biology, Ulm University, James-Franck-Ring, 89069 Ulm, Germany

**Keywords:** Reproduction, Sexual maturation, Aging, RNA-seq, Naked mole-rat, Eusociality

## Abstract

**Background:**

Naked mole-rats (NMRs) are eusocially organized in colonies. Although breeders carry the additional metabolic load of reproduction, they are extremely long-lived and remain fertile throughout their lifespan. This phenomenon contrasts the disposable soma theory of aging stating that organisms can invest their resources either in somatic maintenance, enabling a longer lifespan, or in reproduction, at the cost of longevity. Here, we present a comparative transcriptome analysis of breeders vs. non-breeders of the eusocial, long-lived NMR vs. the polygynous and shorter-lived guinea pig (GP).

**Results:**

Comparative transcriptome analysis of tissue samples from ten organs showed, in contrast to GPs, low levels of differentiation between sexes in adult NMR non-breeders. After transition into breeders, NMR transcriptomes are markedly sex-specific, show pronounced feedback signaling via gonadal steroids, and have similarities to reproductive phenotypes in African cichlid fish, which also exhibit social status changes between dominant and subordinate phenotypes. Further, NMRs show functional enrichment of status-related expression differences associated with aging. Lipid metabolism and oxidative phosphorylation—molecular networks known to be linked to aging—were identified among most affected gene sets. Remarkably and in contrast to GPs, transcriptome patterns associated with longevity are reinforced in NMR breeders.

**Conclusion:**

Our results provide comprehensive and unbiased molecular insights into interspecies differences between NMRs and GPs, both in sexual maturation and in the impact of reproduction on longevity. We present molecular evidence that sexual maturation in NMRs is socially suppressed. In agreement with evolutionary theories of aging in eusocial organisms, we have identified transcriptome patterns in NMR breeders that—in contrast to the disposable soma theory of aging—may slow down aging rates and potentially contribute to their exceptional long life- and healthspan.

**Electronic supplementary material:**

The online version of this article (10.1186/s12915-018-0546-z) contains supplementary material, which is available to authorized users.

## Background

The unique biology of the naked mole-rat (NMR, *Heterocephalus glaber*) has fostered its increasing popularity as an animal model in a variety of research fields. This includes an exceptionally long lifespan and resistance to cancer [1, 2]. The Animal Ageing and Longevity Database (AnAge) [3] cites the maximum recorded lifespan at 31 years, i.e., 368% of the prediction based on body mass. NMRs remain fertile throughout their long and healthy life [4]. This lifelong fertility is even more astonishing given the extreme reproductive skew in NMR colonies. Like eusocial insects, NMRs are socially organized in colonies consisting of a pair of reproducing animals (breeders, queen, and pasha) and up to 300 subordinates (non-breeders, female and male workers) [5]. In principle, workers are capable of reproduction [6, 7], but their sexual maturation is suppressed by the presence of the dominating breeding pair [8–10]. Whether this “social suppression” is caused by “active suppression by breeders,” protective “self-restraint by helpers,” or both, is currently unanswered. Non-breeding animals of both sexes constitute the backbone of the social organization of the colony, with responsibility for foraging, brood care, colony defense, and digging [11].

Naturally, new NMR colonies originate from fissioning of existing colonies or formation of new ones by dispersers who leave their natal colony [12, 13]. When, under laboratory conditions, non-breeders are removed from the colony and paired with the opposite sex, they have the capability to ascend into breeders. This process is accompanied with physiological and behavioral changes and results in the formation of a new colony [5, 6]. As per evolutionary theories of aging in eusocial organisms [14], despite the queen’s enormous metabolic load of producing a large litter every 3 months and being exclusively in charge of lactation [15], data from wild and laboratory NMR colonies indicate that breeders live longer than their non-breeding counterparts [16, 17]. Previously, it was shown that closely related African *Fukomys* mole-rats, which have a eusocial structure similar to NMRs, show lifespan differences between reproductive and non-reproductive animals [18, 19]. Those observations contrast the disposable soma theory of aging. This theory hypothesizes that energy is scarce and can either be invested in maintenance of somatic tissue or in reproduction [20]. Species confronted with a high extrinsic mortality invest their resources in reproduction to ensure survival of the species, at the expense of their lifespan. In the case of low-extrinsic mortality, however, it seems to be more effective to allocate resources to somatic tissue, thereby keeping the body healthy and enabling a longer reproduction during a longer lifespan.

The role of gonadotropin-releasing hormone (GnRH) in mediating environmental cues to allow or block reproduction is well described in a variety of species [21, 22]. In NMR, the reproductive suppression in female non-breeders is mediated through inhibition of GnRH secretion from the hypothalamus [6]. This in turn leads to an inhibition of follicle stimulating hormone (FSH) and luteinizing hormone (LH) released by the pituitary gland, therein blocking ovulation. Reproductive suppression for NMR male non-breeders is also caused by inhibition of GnRH secretion, in this case resulting in lower levels of urinary testosterone and plasma LH [7]. However, the impact is less profound compared to females as spermatogenesis is attenuated, but not entirely suppressed [23]. Nevertheless, weight of testis and number of active spermatozoa are higher in breeders [7, 24].

The NMR can be regarded as a neotenic species, and the prolonged retention of juvenile features has been linked to its longevity [25]. In comparison to mice, e.g., postnatal NMR, brain maturation occurs at a slower rate [15] and puberty is delayed. Female and male NMRs may reach sexual maturity at 7.5 to 12 months of age [26]. In the colony, however, the presence of queen and pasha leads to suppression of sexual maturation in both non-breeding males and females [8–10] and can delay—independently of neoteny—the puberty of female workers throughout life [27]. Sexual dimorphism is almost absent among non-breeding NMRs [28, 29]; both sexes show almost no difference in morphology—including body mass, body size, and even external genitalia—as well as no behavioral differences, in the sense that non-breeders participate and behave equally in all colony labors [30]. Nevertheless, these features are correlated with colony rank. The most profound differences can be observed by comparing NMR queens vs. non-breeders and are reflected in morphological differences, such as the elongated spine and higher body mass of queens, and behavioral differences, such as increased aggressiveness, copulation, and genital nuzzling [30].

In this work, we have characterized the transcript signature of reproductive status (breeder vs. non-breeder) in tissue samples of ten organs or their substructures (hereinafter referred to as “tissues”), from both sexes, using RNA-seq. We have contrasted the NMR results with the transcript profiles of corresponding samples of guinea pig (GP, *Cavia porcellus*). The GP is a closely related, social polygynous rodent in which normally all adult members of large social groups contribute to reproduction; females have spontaneous ovulation and postpartum estrus [31]. Contrary to the NMR, the GP is characterized as a not long-lived rodent species (AnAge: 12 years maximal longevity, 89% of the prediction based on body mass). We specifically focused our analyses on transcriptome signatures of the sexually dormant NMR non-breeders (workers) and the differentially expressed genes (DEGs) that may contribute to the exceptional long life- and healthspan of NMR breeders. In agreement with evolutionary theories of aging, we identified transcriptome patterns in breeders that contrast the disposable soma theory of aging and may contribute to NMR long life- and healthspan.

## Results

To gain molecular insights into the fascinating combination of NMR phenotypes, in particular, their eusocial reproduction, lifelong fertility, extraordinary healthspan, and longevity, we aimed to collect a comprehensive set of tissues for male and female breeders and non-breeders of NMR and GP—six biological replicates each. Towards this, NMR non-breeders were removed from their natal colony, paired with an unrelated partner of the opposite sex from a second colony and thereby turned into breeders. The transformation process into successful breeders took 118.5 ± 138.6 days (average ± SD, range 10–382 days). Respective male and female litter siblings remained in the two colonies as non-breeder controls. Time to first litters averaged in 6.5 ± 4.9 months and duration of pregnancies was approximately 70 days. Serving for comparison, GP is the closest related not long-lived rodent species (92.1% identity of protein-coding sequence; Additional file 1: Figure S1) for which a genome sequence is available. GP breeders and non-breeders were housed as pairs of opposite or same sex, respectively. For this species, the time to first litters was 4.1 ± 0.8 months, and the pregnancies lasted about 68 days.

Female breeders gave birth to two litters each, with two exceptions. One NMR female was pregnant at least twice (ultrasonographically verified), but never gave birth to live offspring, and another gave birth to three litters, due to a pregnancy fathered by one of her sons. At time of sampling, NMRs and GPs reached an age of 3.4 ± 0.5 and 0.9 ± 0.1 years, respectively (Additional file 2: Table S1). Tissue samples were collected after the second litters were raised and last lactation has stopped (421.0 ± 141.6 days, range 274–678 days). The average NMR colony size at that point was 17.7 ± 3.8 (range 12–24). Thus, taking into account the usual half-life of a transcript and protein of hours to weeks [32], the initial stressors caused by the isolation of the breeders in the beginning of the experiment are very unlikely to affect the transcriptome data obtained months and even years later.

### Tissue and species are the major determinants of transcriptomes

To compare gene expression between reproductive statuses (breeder vs. non-breeder) in NMR and GP, we performed RNA-seq of ten different tissues (heart—Hrt, skin—Skn, liver—Lvr, kidney—Kid, cerebellum—Cer, hypothalamus—Hyp, pituitary—Pit, thyroid—Thy, adrenal—Adr, and gonads—Gon, represented by either ovary—Ova or testis—Tes) from 24 animals for each species (six males, six females per status; Additional file 1: Figure S2). The tissues were chosen to represent sexual maturation and reproduction (Gon), the endocrine and stress response system involved in social status definition (Hyp, Pit, Thy, Adr), and main organs affected by aging (Hrt, Skn, Lvr, Kid, Cer). Seven of the 480 samples (1.5%) had to be excluded for different reasons (Additional file 2: Tables S2, S3). On average ± SD, we obtained per sample 27.6 ± 3.6 million high-quality reads with 84.1 ± 16.1% unique mapping rate (Additional file 2: Table S4). The grand mean of pairwise Pearson correlation within the 40 replicate groups (2 statuses × 2 sexes × 10 tissues per sex) was 0.981 ± 0.013 and 0.984 ± 0.01 for NMR and GP, respectively, indicating high consistency between replicate samples (Additional file 2: Table S5).

**Fig. 1:**
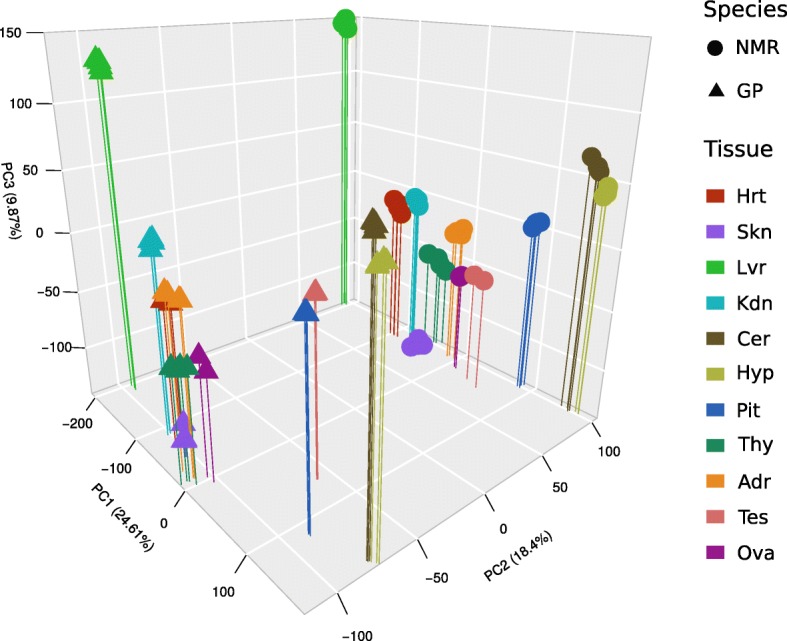
Principle component (PC) analysis of groups based on mean expression levels (four groups per tissue and species: 2 sexes × 2 statuses, except gonads; raw data provided in Additional file 2: Table S6). Tissues are separated by PC1 and PC3, species by PC2

Based on these data, unsupervised hierarchical clustering gave a similar cluster hierarchy of tissues for both species (Additional file 1: Figure S3). Brain tissues are grouped (Pit as a sister group to Cer and Hyp); Kid and Thy are sister groups to the cluster of Adr and Ova. The results are confirmed by principle component analysis, separating tissues by the first and species by the second component (Fig. 1; Additional file 2: Table S6). At this level of analysis, ovary was the only tissue, which showed a separation of samples with respect to breeding status. Together, this indicates that (i) tissue source is dominant over other biological variables such as species, sex, and status; and (ii) the impact of sex and status on transcriptome profiles is subtle.

### Cross-species DEGs are enriched in aging-related genes

To further characterize the species differences between the long-lived NMR and the shorter-lived GP, we determined gene expression differences based on orthologous transcribed regions that show high sequence similarity. This filtering method avoided potentially misleading signals that may arise from assembly artifacts or the comparison of different transcript isoforms and identified 10,127 genes suitable for further analyses.

**Fig. 2:**
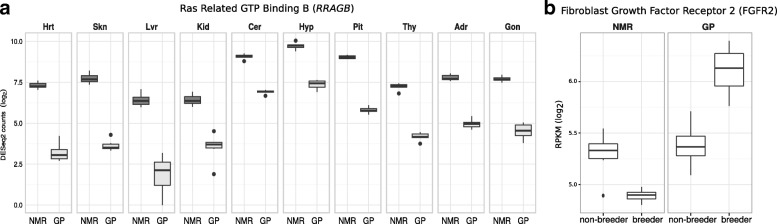
**a** Ras related GTP binding B (RRAGB) is consistently differentially expressed between species across all tissues. RRAGB is known to interact with mTORC1 complex [33, 34]. **b** Fibroblast Growth Factor Receptor 2 (FGFR2) shows opposing direction of expression between NMR and GP in breeders vs. non-breeders

Across all tissues, we identified 18,000 significant expression differences (EDs; 9651/8349 higher/lower expressed in NMR) in 5951 out of 10,127 genes (FDR < 0.01, |log_2_FC| > 2; Additional file2: Table S7, Additional file 3: Tables S1–S11). Among genes that are differentially expressed across all tissues (29 DEGs; Additional file 2: Table S8), we identified aging-related candidates that show consistent direction of expression change. For example, *RRAGB* (Ras related GTP binding B, Fig. 2a) and *TMEM8C* (transmembrane protein 8C) are higher expressed in NMRs. *RRAGB* interacts with mTORC1 complex [33, 34]. *TMEM8C* is essential for muscle regeneration [35] and might be linked to the resistance to muscle loss in aged NMRs [36, 37]. On the other side, among DEGs showing higher expression in GPs, we identified PLK4 (Polo-like kinase 4). PLK4 is involved in cell cycle and localizes to the centriole. Interestingly, overexpression of PLK4 mRNA has been observed in several cancer types [38].

To further assess the association of cross-species DEGs with aging, we examined their overlap with aging-related genes of human and mouse obtained from the Digital Ageing Atlas (DAA) [39]. This test revealed a significant overlap with DAA containing 1056 genes (17.74% of DEGs; *p* = 0.006, Fisher’s exact test (FET); Additional file 2: Table S9). The enrichment analysis of shared aging-related genes (Additional file 2: Table S10) reveals that the top-ranked GO term set is associated with lipid biosynthetic process (GO:0008610) (Additional file 1: Figure S4), a finding referring to existing links between lipid metabolism and lifespan [40, 41].

### Sexual differentiation and maturation in NMR are delayed until transition from worker to breeder

**Fig. 3:**
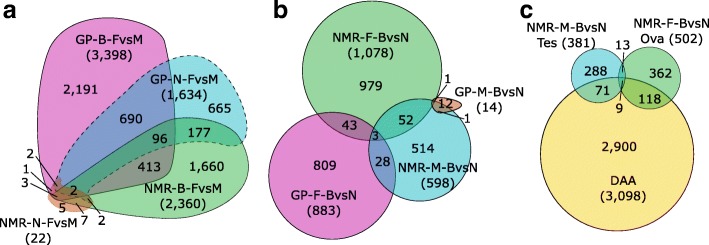
Euler diagrams showing overlaps of DEGs. **a** Female vs. male. **b** Breeder vs. non-breeder. **c** Gonads of NMR breeder vs. non-breeder and aging-related genes from the Digital Ageing Atlas (DAA)

The DEGs between sexes were determined within the groups of non-breeders and breeders for each tissue and species (Table 1, Additional file 2: Table S11, Additional file 4: Tables S12–S47). GP non-breeder females vs. males (GP-N-FvM) show over all tissues except gonads 1713 significant EDs in 1634 genes (FDR < 0.01, Table 1), primarily in Adr (858 DEGs), Lvr (383), Thy (347), and Kid (109). Between female and male GP breeders (GP-B-FvM), 3654/3398 EDs/DEGs were observed. These transcriptome data confirm a clear sexual differentiation among sexually mature GPs that further increases after onset of breeding. Breeders have 790 DEGs in common with non-breeders (*p* < 2.2 × 10^−16^, FET; Fig. 3a). Functional enrichment analysis of these shared genes reveals among the highest ranked GO sets immune system-related terms (Additional file 1: Figure S5, Additional file 2: Table S12).

**Table 1:** Numbers of DEGS identified in the different comparisons (FDR < 0.01)

Tissue	Female vs. male				Breeder vs. non-breeder			
	Non-breeder		Breeder		Females		Males	
	GP	NMR	GP	NMR	GP	NMR	GP	NMR
Hrt	4	10	89	13	2	3	0	0
Skn	6	6	3	5	5	1	0	223
Lvr	383	4	235	10	71	0	9	1
Kid	109	6	106	21	1	4	0	0
Cer	1	5	2	25	0	15	0	0
Hyp	2	8	1	11	0	5	2	0
Pit	347	9	307	47	114	114	2	1
Thy	3	0	2087	1791	675	285	1	0
Adr	858	4	824	533	0	201	1	4
Gon	–	–	–	–	18	502	3	381
ED*	1713	52	3654	2456	886	1130	18	610
NR†	1634	22	3398	2360	883	1078	14	598

*Significant expression differences across tissues

†Non-redundant set of significant expression differences across tissues

Similar to GP-B-FvM, NMR-B-FvM showed 2456/2360 sex-related EDs/DEGs (FDR < 0.01), mostly in Thy (1791) and Adr (533). The overlap with GP-B-FvM is with 514 DEGs considerable but does not reach significance (*p* = 0.062, FET; Fig. 3a). Nevertheless, these data indicate basic similarities in sexual differentiation among breeders of both species.

Surprisingly, only 22 NMR-N-FvM EDs/DEGs were detected across all tissues (Table 1, Additional file 2: Table S13), indicating an only minor sex differentiation between NMR non-breeding females and males on the transcriptional level, consistent with the almost absent sexual dimorphism among non-breeding NMRs [28, 29].

### Status change of NMRs is accompanied by major changes in the endocrine system

The DEGs between breeders and non-breeders were determined within the same sex for each species (Table 1; Additional file 2: Table S14, Additional file 5: Table S48–S87). Females showed a similar amount of EDs/DEGs in both species (GP-F-BvN: 886/883, NMR-F-BvN: 1130/1078) but have only 46 DEGs in common (Fig. 3b). This is less than expected by chance, although not reaching significance (*p* = 0.075, FET for depletion), and indicates that the molecular signature of the transition from female non-breeder to breeder is different in both species. For example, in GP-F-BvN, only 18 DEGs are observed in Ova and none in Adr, while in NMR-F-BvN, these tissues show most of the differences with 502 and 201 DEGs, respectively. Functional enrichment analysis of DEGs in NMR Ova identifies reproductive structure development (GO:0048608) as the highest ranked category (Additional file 1: Figure S6, Additional file 2: Table S15). The same analysis in Adr revealed an obvious directionality in expression changes. DEGs are preferentially upregulated among highest ranked GO term sets (Additional file 1: Figure S7, Additional file 2: Table S16), e.g., in reproduction (GO:0000003, 24 of 28) and endocrine system development (GO:0035270, 17 of 18). Tallying with this, Cer DEGs are enriched and upregulated in steroid metabolic process (GO:0008202, 6 of 6 upregulated) and response to hormones (GO:0009725, 7 of 7) (Additional file 1: Figure S8, Additional file 2: Table S17).

In male GPs, status-related differences (GP-M-BvN) were almost absent across all tissues (only 18 EDs/14 DEGs, Table 1). In contrast, NMR-M-BvN showed 610/598 EDs/DEGs, predominantly in Tes (381) and Skn (223). NMRs share 55 status-related DEGs in both sexes (*p* = 0.008, FET; Additional file 2: Table S18), while the few status-related changes in male GPs showed no overlap with those in females (Fig. 3b). Among shared DEGs in NMRs, 10 genes involved in endocrine signaling were identified, including *SSTR3* (somatostatin receptor), *TAC4* (tachykinin), *PRDX1* (peroxiredoxin 1), and ACPP (acid phosphatase, prostate), as well as in general signaling via cAMP signaling (three genes) and through G-protein coupled receptors (four genes) further underlining that social status transition in NMR is associated with changes in the endocrine system.

### Mitochondrial genes show opposed expression changes in Tes and Skn after status change of NMR males

In NMR-M-BvN Tes, functional enrichment analysis revealed as highest ranked metabolism- and energy-related GO sets. DEGs enriched therein are mostly upregulated, e.g., lipid biosynthetic process (GO:0008610, 75 of 82 genes) and oxidation-reduction process (GO:0055114, 64 of 64) (Additional file 1: Figure S9, Additional file 2: Table S19). Furthermore, we observed an upregulation of response to stimulus (GO:0050896, 79 of 106), in line with an upregulation of steroid metabolic process (GO:0008202, 28 of 28) included in lipid biosynthetic process set. In accordance with the dominance of energy-related processes, DEGs are enriched and preferentially upregulated in the top GO cellular component terms mitochondria (GO:0005739, 61 of 62) and peroxisomes (GO:0005777, 13 of 13) (Additional file 2: Table S20). Together, this indicates increased demands of energy, e.g., to produce steroid hormones in Tes of NMR breeders.

Similar to Tes, Skn showed enrichment of energy-related processes (Additional file 2: Table S21, Additional file 1: Figure S10). However, GO term sets in Skn are mostly downregulated, including energy derivation by organic compounds (GO:0015980, 36 of 38 genes) and oxidation-reduction process (GO:0055114, 42 of 46). Consistently, this includes genes associated with mitochondria (GO:0044429, 56 of 57) and respiratory chain (GO:0070469, 11 of 11).

**Fig. 4:**
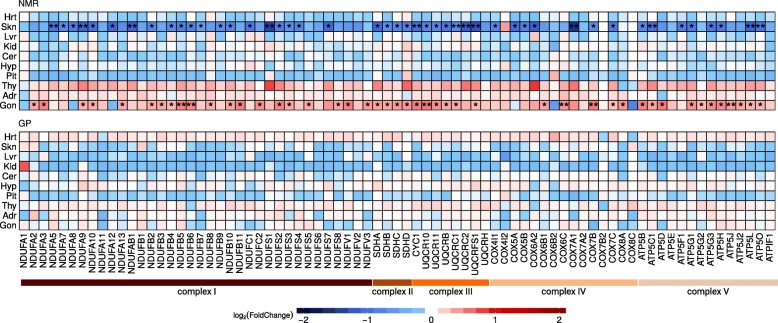
Expression changes of nuclear genes encoding for mitochondrial respiratory chain complexes in status change of male NMRs (top) and GPs (bottom). DEGs are indicated by asterisk (*FDR < 0.05, **FDR < 0.01). Only NMRs show significant expression differences (skn 46 genes, mean fold-change 0.76; tes 46 genes, mean fold-change 1.89)

The overlap between mitochondrial DEGs in Tes and Skn comprises six genes (*p* = 7.27 × 10^−8^, FET; Fig. 4). Among common genes, *PINK1* (PTEN induced putative kinase 1) is 1.5-fold upregulated in Tes and 2.5-fold downregulated in Skn, indicating a role in regulation of mitophagy [42] in both tissues.

To follow up the mitochondria-related findings, a “mitonuclear ratio” was determined as the RNA-seq read count ratio of mitochondrial-encoded genes versus nuclear-encoded genes. It differs largely between tissues and species (Additional file 2: Table S22). Hrt showed the highest mitonuclear ratio, with a minor difference between species (NMR 30.1%, GP 30.4%). Tes showed the lowest ratio, particularly in GPs (NMR 5.5%, GP 0.9%), and a 43.1% increase in NMR-M-BvN (Additional file 1: Figure S11). This increase is accompanied with an upregulation of nuclear genes encoding for mitochondrial respiratory chain complexes (Fig. 4; Additional file 2: Table S23). The expected increase in ROS is compensated by an on average 1.59-fold upregulation of eight antioxidant DEGs (Additional file 2: Table S24). Consistent with functional enrichment analysis mentioned above, an opposing effect in Skn of NMR male breeders was observed, which showed a decline in mitonuclear ratio together with a downregulation of nuclear genes of the respiratory complexes (Fig. 4). In line with the downregulation of the oxidative phosphorylation pathway (OXPHOS), a downregulation of antioxidant enzymes SOD2 (superoxide dismutase 2, 2.64-fold) and PRDX3 (peroxiredoxin 3, 2.14-fold) was observed. In general, downregulation of OXPHOS is linked to an extended lifespan [40, 43–45].

### NMR status-related DEGs are enriched in aging-related genes

Guided by the exceptional long life- and healthspan of NMR breeders, we searched our transcriptome data for molecular signatures relating to this phenomenon. First, we found that only NMRs show status-related DEGs that are significantly enriched for aging-related genes from DAA (Additional file 2: Table S25): males in Skn (55 genes; *q* = 0.0012, FET, *q* refers to *p* values adjusted for multiple testing) and Tes (80 genes, *q* = 0.01), and females in Ova (127 genes; *q* = 1.2 × 10^−7^), Thy (59 genes, *q* = 0.033), and Adr (43 genes, *q* = 0.038). The significant overlap of 22 DEGs between NMR-F-BvN and NMR-M-BvN in Gon (*p* = 0.0035, FET) contains nine aging-related genes (*p* = 0.004, Fig. 3c). In GP, only the non-redundant set of DEGs in GP-F-BvN showed a tendency of enrichment (160 genes, *q* = 0.051), in contrast to NMRs, which showed enrichment in males (134 genes, *q* = 4.5 × 10^−5^) and females (245 genes, *q* = 7 × 10^−9^).

Second, we hypothesized that reproduction impacts the life expectancy of NMR and GP differently. Therefore, we searched for status-related DEGs that are shared in both species but show opposing direction of expression. These genes might mark different coping mechanisms with the metabolic load of reproduction. As described above, the overlap of DEGs between species is very low (Additional file 2: Table S26). Nevertheless, opposing direction of expression change can be observed in Ova (1 of 2 shared DEGs) and female thyroid (8/8) and testis (1/1). For example, the fibroblast growth factor receptor 2 gene (*FGFR2*), linked to aging (AgeFactDB) [46], is in Ova downregulated in NMRs, but upregulated in GPs (Fig. 2b).

Third, and based on the assumption that status-related EDs with the greatest interspecies difference (regardless of directionality) have an impact also on species-specific aging trajectories of breeders, we determined enrichment of aging-related genes in the upper quintile of those genes (Additional file 2: Table S27). In males, we found significant enrichments of DAA genes in Skn (*q* = 7.04 × 10^− 7^) and Tes (*q* = 0.0025), in females in Skn (*q* = 0.0064), Hrt (*q* = 0.0024), Pit (*q* = 0.0053), and Ova (*q* = 0.0013). Further functional enrichment analysis of these aging-related gene sets reveals differences between sexes. In males, the non-redundant set of genes shows enrichment for lipid metabolism (GO:0006629), energy metabolism (energy derivation by oxidation of organic compounds, GO:0015980; mitochondrial ATP synthesis coupled proton transport, GO:0042776), glutathione metabolic process (GO:0006749), and immune system (innate immune response-activating signal transduction, GO:0002758) (Additional file 2: Table S28, Additional file 1: Figure S12). Females showed enrichment in positive regulation of tumor necrosis factor production (GO:0032760) and negative regulation of programmed cell death (GO:0043069) (Additional file 1: Figure S13, Additional file 2: Table S29).

### Status-related changes in NMR contradict the disposable soma theory

**Fig. 5:**
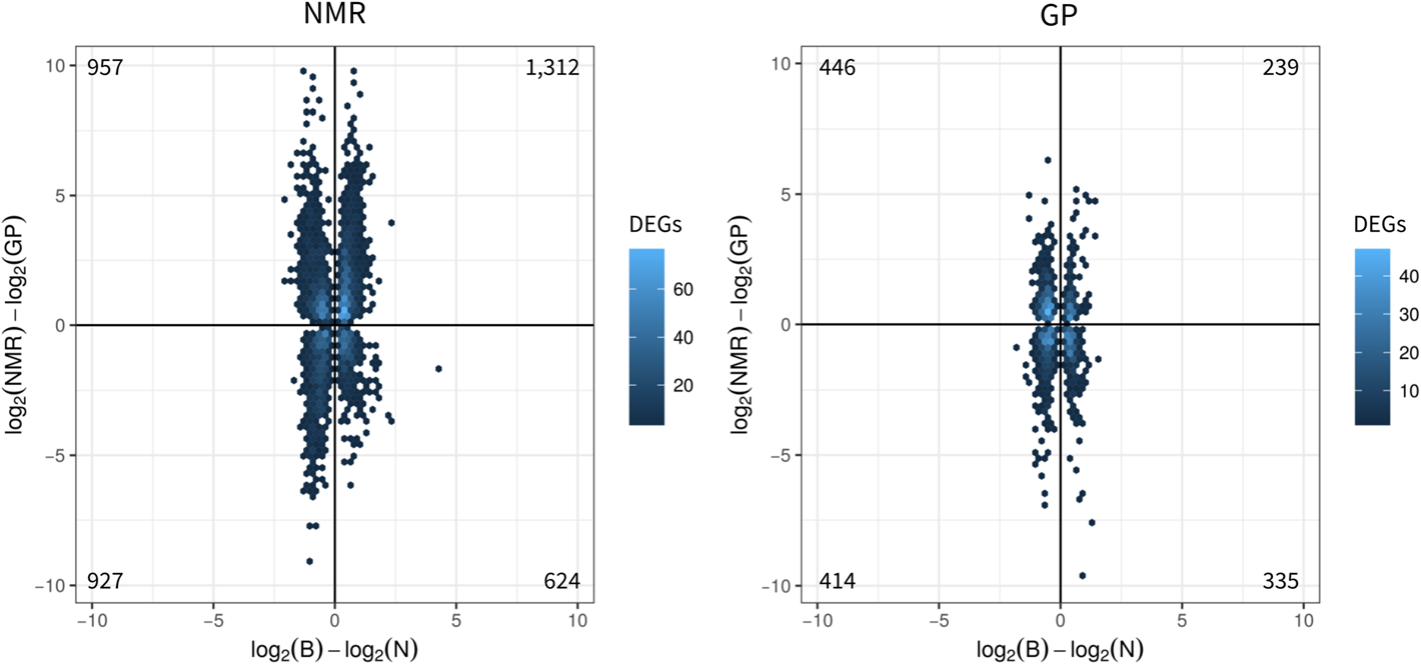
DEGs (FDR < 0.05) occurring in both comparisons: cross-species (NMR vs. GP, *y*-axes) and status change GPs (breeder vs. non-breeder, *x*-axes) separately for NMRs and GP. Correlation analysis between species shows opposing correlation (Lancaster procedure [47], *p* = 4.2 × 10^−9^), while status-related DEGs in NMR are positively correlated with cross-species DEGs (DEGs = 3820; spearman correlation = 0.17, *p* = 3.2 × 10^−27^), status-related DEGs in GP show a negative correlation (1434; −0.1; 1.8 × 10^− 4^)

Moreover, we assessed the connection of cross-species DEGs with expression changes that are associated with status change in each species. Based on DEGs shared in two comparisons (NMR vs. GP and breeder vs. non-breeder, FDR < 0.05), we performed correlation analyses of fold changes in each species. We hypothesized—in agreement with the disposable soma theory of aging—a negative impact of reproduction on lifespan for GP and—in contradiction to this theory—an inverse effect for NMR. This was confirmed by opposing correlations (combined *p* = 4.2 × 10^−9^) (Lancaster procedure [47]), negative correlation for GP and positive correlation for NMR (Fig. 5). This means that DEGs with higher expression in NMR than GP are preferentially upregulated in NMR breeders compared to non-breeders, and vice versa; in other words, a transcriptome pattern associated with longevity (NMR vs. GP) is reinforced by the status transition (breeder vs. non-breeder) and thus may in agreement with evolutionary theories of aging in eusocial organisms contribute to the exceptional long life- and healthspan of NMR breeders.

## Discussion

Our comparative study of breeders vs. non-breeders of the eusocial long-lived NMR and the polygynous and shorter-lived GP has afforded us a comprehensive set of transcriptome data, providing insights into molecular networks underlying naturally evolved interspecies differences in sexual maturation and links between reproduction and aging. Both species can breed year-round and produce four to five litters per year [48, 49]. Both have a similar average gestation period of ~ 70 days, which is long compared to similarly sized species. Notably, NMRs produce on average 10.5 offspring per litter—twice the number produced by similarly sized rodents and over three times higher than GPs (average 3.2 offspring) [48, 49]. This underscores the apparent contradiction of the NMR queen’s enormous metabolic load and extraordinarily long life- and healthspan [4] to the disposable soma theory of aging [20], indicating that a natural means to extended healthspan remains to be uncovered in NMRs.

A first study to identify adaptations to unique NMR traits at the transcriptome level compared liver gene expression of young adult non-breeding male NMRs and mice [50]. Higher NMR transcript levels were observed for genes associated with oxidoreduction and mitochondria. This present study, more comprehensive in several aspects (sex, breeding status, numbers of animals, and tissue samples), is based on a comparison of NMR vs. GP, which are phylogenetically closer than NMR and mouse (Additional file 1: Figure 1). It revealed that between NMR and GP, 58.8% of the analyzed genes are differentially expressed and that these DEGs are significantly enriched for aging-related genes. Among the latter, the main functional commonality is their association with lipid metabolism. Links of this molecular network to longevity of NMR were also obtained by a parallel proteome comparison of NMR and GP livers [51]. This study also shows that NMR liver mitochondria exhibit an increased capacity to utilize fatty acids.

In respect of sex-specific molecular signatures among either breeders or non-breeders in NMR and GP, the main finding is a near complete absence of significant transcriptional differences between sexes in non-breeding NMRs. This is in stark contrast to non-breeding GPs of an even younger age, where we observed more than a thousand DEGs, and fits the grossly identical morphology and identical behavior of non-breeding NMR’s in stable colonies [30]. GP non-breeders and breeders share a large and highly significant number of sex-related DEGs. These DEGs, among others, are enriched in GO terms related to steroid metabolism and immune system. The effect of gonadal steroids on the immune system is well described in GPs and other mammals [52]. After separation of NMR non-breeders from their colony, sexes became not only distinguishable by morphology and behaviour [27, 30], but also by gene expression. This differentiation on transcriptional level provides further molecular support for the previously described suppression of sexual maturation in non-breeding adult NMRs by social stress [8, 27] and identified major changes in the endocrine system after status change in NMRs, but not in GPs (Additional file 6: Text S1). Notably, we found no significant differences in gonadotropin-related genes (Additional file 6: Text S2), indicating similar transcript turnovers in both non-breeders and breeders—in line with previous results indicating that LH is stored in non-breeders, ready to be released upon GnRH signaling [53].

Glucocorticoids have been linked to stress, reproduction, and social behavior in a variety of species, including members of muroidae, primates, and cichlids [54–56]. In NMRs, however, correlation between social status and urinary cortisol is not clear and seems to depend on colony stability [57, 58]. Here, we observed a significant upregulation of *NR3C1* (glucocorticoid receptor) in Tes of NMR male and Thy of female breeders (Additional file 4: Tables S55,S67). Interestingly, this is in line with elevated expression of glucocorticoid receptor in Tes in African cichlid breeders, where males can reversibly change between dominant and subordinate phenotypes [59]. Similar to NMRs, only dominant phenotypes are reproductively active. Moreover, in male African cichlids, aggression is negatively correlated with expression of *SSTR3* (somatostatin receptor 3) in Tes. Similarly, we observed significant downregulation of *SSTR3* in NMR breeder Tes (Additional file 5: Table S67). This indicates that *SSTR3* may also be associated with social dominance in NMRs.

As our study was primarily motivated by the exceptional long life- and healthspan of NMR breeders, we sought evidence indicating that NMR status change has an impact on genes involved in aging. We found enrichment of aging-related genes in the non-redundant DEG sets of males and females, as well as enrichment in most tissues showing at least 50 DEGs (male Skn and Tes; female Ova, Thy, and Adr). This contrasts with our observations in GPs, which showed only a tendency of aging relation for the non-redundant set of status-related DEGs in females.

We further observed significant tissue-specific changes in OXPHOS of male NMR breeders. While Tes showed an upregulation of nuclear-encoded mitochondrial genes and a respective increase in mitonuclear transcript ratio, Skn showed the opposite. Significant enrichments of genes involved in fatty acid metabolism among status-related DEGs in both NMR tissues were also noted. Consistent with the role of mitochondria in lipid homeostasis and the observed directionality of changes in OXPHOS, fatty acid metabolism DEGs in Tes were preferentially upregulated and in Skn, downregulated. While the increased mitochondrial activity in Tes probably complies with demands of energy for the production of sex steroids and their anabolic effect on physiology, such as growth of testis [24], the observed changes in Skn may indicate a link to the extraordinary healthspan of male NMR breeders. It has previously been observed that inhibition of complex I activity during adult life prolongs lifespan and rejuvenates the tailfin transcriptome in short-lived fish [44]. Enhanced lipid metabolism and reduced mitochondrial respiration were also linked to NMR longevity in a parallel liver proteome study comparing NMR vs. GP and old vs. young NMR [51]. From nematodes to humans, it has been shown that lifespans differ between sexes and that aging is a sex- and tissue-specific process [60–63]. In agreement therewith, this potential mechanism to slow down Skn aging rates was only observed in male NMRs.

Finally, we performed a correlation analysis between species (NMR vs. GP) and status (breeder vs. non-breeder) EDs, confirming the basic hypothesis of the present work: in contrast to GP and in line with recent demographic studies performed in NMRs [17] and with evolutionary theories of aging in eusocial organisms, the transition into breeders results in molecular signatures linked to extended life- and healthspan only in NMRs. Genes which are higher or lower expressed in NMR compared to GP are also preferentially up- or downregulated in NMR breeders (positive correlation), opposite to GPs (negative correlation). In other words, the positive correlation in NMR contradicts the disposable soma theory of aging, as EDs contributing to a long lifespan (higher/lower expression in NMR than GP) are preferentially increased in NMR breeders compared to non-breeders, while diminished in GPs—as suggested by this theory.

## Conclusion

Taken together, our comparative transcriptome analysis of breeders vs. non-breeders of the eusocial, long-lived NMR vs. the polygynous and shorter-lived GP identifies molecular networks underlying socially regulated sexual maturation and naturally evolved extended life- and healthspan that encourages further functional and mechanistic investigations of these extraordinary NMR phenotypes.

## Methods

### Animals

#### Naked mole-rats

NMR colonies were kept inside a climatized box (2 × 1 × 1 m) in artificial burrow systems, consisting of eight cylindrical acrylic glass containers (diameter 240 mm, height 285 or 205 mm). The latter functioned as variable nest boxes, food chambers, or toilet areas, and were interconnected with acrylic tubes having an inner diameter of 60 mm. Husbandry conditions were stabile during the entire experimental period of 22 months. Temperature and humidity were adjusted to 27.0 ± 2.0 °C and 85.0 ± 5.0%, respectively. In general, the NMR colonies were kept in darkness except for 2 to 4 h of daily husbandry activities. Fresh vegetable food was provided daily and ad libitum. In addition, commercial rat pellets (Vita special, Vitakraft GmbH, Bremen, Germany) were fed as an additional source of protein and trace elements.

To turn them into breeders, randomly selected non-breeding animals derived from two long-term (> 4 years) established colonies of more than 50 individuals were separated and paired with the opposite sex. As non-breeder controls, litter siblings of paired animals remained in their colonies as workers. After the lactation period of the second set of live offspring, the tissue sampling was scheduled. To avoid further pregnancies in the females, male partners were removed and euthanized 8–10 days postpartum. The tissue collection in the females took place 40–50 days after the end of last pregnancy.

#### Guinea pigs

GPs (breed: Dunkin Hartley HsdDhl:DH, Harlan Laboratories, AN Venray, Netherlands) were housed in standardized GP cages (length 850 mm, width 470 mm, height 450 mm) in breeding pairs plus offspring or in same-sex pairs of two. Commercial guinea pig pellets and commercial pet food hay (Hellweg Zooland GmbH, Berlin, Germany) were provided together with vitamin C-enriched water ad libitum. Housing temperature and humidity were 18.0 ± 2.0 °C and 45.0 ± 5.0%, respectively. A 12-h light/dark regime was provided.

After an initial adaption period of 6 to 8 weeks, the GPs were randomly divided in breeding pairs or in same-sex pairs of two. The offspring were separated from their parents after weaning (~ 3 weeks postpartum). Tissue collection was scheduled after the lactation period of the second set of live offspring. To avoid further pregnancies in the females, male partners were removed between 11 days before and 7 days after birth of the second litter. The tissue collection in the females took place 42–83 days after the end of last pregnancy.

For tissue collection, all animals were anesthetized by 3% isoflurane inhalation anesthesia (Isofluran CP, CP-Pharma, Burgdorf, Germany) and euthanized by surgical decapitation.

### Sample collection, RNA isolation, and sequencing

For de novo transcriptome assembly, animals were euthanized and ten tissue samples (heart—Hrt (NMR only), skin—Skn, liver—Lvr, kidney—Kid, cerebellum—Cer, hypothalamus—Hyp, pituitary—Pit, thyroid—Thy, adrenal—Adr, and gonads—Gon (testis—Tes /ovaries—Ova)) were collected from NMR and GP individuals, as described previously [64]. Strand-specific RNA-seq were prepared using the TruSeq Stranded RNA LT Kit (Illumina), and 200-nt reads were obtained using a HiSeq2500 (Illumina), as described previously [64].

For expression analysis, the same ten tissues were collected from NMR and GP breeders and non-breeders. RNA was purified as described above. Library preparation was done using Illumina’s TruSeq RNA Library Prep Kit v2 kit following the manufacturer’s description. Quantification and quality check of the libraries was done using Agilent’s Bioanalyzer 2100 in combination with a DNA 7500 Kit (both Agilent Technologies). Sequencing was done on a HiSeq 2500 running the machine in 51 cycle, single-end, high-output mode by multiplexing seven samples per lane. Demultiplexing and extraction of read information in FastQ format was done using the tool bcl2astq v1.8.4 (provided by Illumina).

### Data analysis

De novo transcriptome assembly and annotation for GP was performed as described in [64]. Briefly, overlapping paired-end reads were joined into single fragments and then assembled by Trinity [65]. Gene symbols were assigned to the assembled transcripts by similarity to human transcripts using FRAMA [64].

As a reference for RNA-seq data mapping, the public NMR (Bioproject PRJNA72441) [66] and GP genomes (UCSC, cavpor3) were used. Reference transcript sets of NMR and GP were mapped to the corresponding genome in two steps: BLAT (v36) [67] was used to identify the locus and then SPLIGN (v1.39.8) [68] was applied to splice align the transcript sequence within BLAT locus. RNA-seq data were aligned to the corresponding reference genome utilizing STAR (v2.4.1d) [69] with a maximum mismatch of 6% and a minimum aligned length of 90%. Reads mapped to multiple loci were discarded. Gene expression was quantified using HTSEQ (v0.6.1p1) [70] based on the aligned reference transcripts (Additional files 7 and 8). The pairwise Pearson correlation between biological replicates was calculated based on 16,339 and 16,009 genes in NMR and GP, respectively (Additional file 2: Table S5).

The “mitonuclear transcript ratio” was calculated as the read count ratio of 13 mitochondrial-encoded genes vs. all nuclear-encoded genes.

PosiGene was applied to the transcriptome of human, NMR, and GP with the parameter “-prank=0 -max_anchor_gaps_hard=100 -rs=NMR” to determine orthologous transcribed regions in NMR and GP having a protein identity > 70%. RNA-seq data were aligned to the corresponding transcriptomes utilizing bowtie2 (2.2.9) [71] with the parameter “-very-sensitive-local.”

DESeq2 (v1.6.3) [72] was used to identify DEGs. For each comparison, *p* values were corrected for multiple testing using FDR (Benjamini Hochberg corrected [73]), and a significance level of FDR < 0.01 was used. Across tissues, we distinguish in our terminology between “expression differences” (EDs) and “differentially expressed genes” (DEGs) to indicate the total number (sum; EDs) and the non-redundant set (union; DEGs), respectively, of significant expression changes.

Gene Ontology analyses were performed using the web interface of GoMiner (Database build 2011-01) based on the functional annotation of human genes (UniProt) [74]. Again, we corrected for multiple testing and used a significance level of FDR < 0.05. REVIGO (parameter SimRel = 0.5) was used to summarize results into non-redundant GO term sets [75]. GO term sets were then ranked by number of summarized GO terms and number of changed genes. KEGG analysis was performed using Fisher’s exact test, and significant pathways were identified after multiple testing correction using FDR < 0.05. KEGG results were redundant to Gene Ontology analysis and therefore not shown.

Overlap between gene sets was determined with Fisher’s exact test (FET) using the one-sided option. Generally, we tested for enrichment if not stated otherwise.

We obtained 3009 aging-related genes in human and mouse from the Digital Ageing Atlas (DAA) [39]. The corresponding counterparts in the NMR (2588) and GP (2539) were used for enrichment analysis, and results were corrected for multiple testing (FDR). *P* values corrected for multiple testing are indicated by *q* and nominal *p* values by *p*.

To examine the connection between reproduction and aging in both species, we determined the difference in log2-fold-change (breeders vs. non-breeders) of NMR and GP. For fold-changes moving in opposite directions between species, we calculated the absolute difference (| log_2_*NMR*_*BvsN*_ − log_2_*GP*_*BvsN*_ |), and for fold-changes moving in the same direction, higher fold-changes in NMR-BvN were rewarded (|log_2_*NMR*_*BvsN*_ | − |log_2_*GP*_*BvsN*_|). The 20% quantile of genes having the greatest difference was determined separately for (i) the complete gene set and for genes showing (ii) opposing and (iii) unidirectional fold-changes. All sets were tested for enrichment of aging-related genes.

Statistical analyses were performed in R (version v3.1.2).

## Additional files

Additional file 1: **Figure S1.** Identity of protein-coding sequences between human, mouse, GP, and NMR. **Figure S2.** Schema of collected tissues. **Figure S3.** Hierarchical clustering of gene expression profiles. **Figure S4.** Top 15 highest ranked GO sets based on enrichment analysis of cross-species DEGs between NMR and GP. **Figure S5.** Top 15 highest ranked GO sets based on enrichment analysis of sex-related DEGs that are shared between GP breeder and non-breeder. **Figure S6.** Top 15 highest ranked GO sets based on enrichment analysis of status-related DEGs in the ovary of NMR females. **Figure S7.** Top 15 highest ranked GO sets based on enrichment analysis of status-related DEGs in adrenal gland of NMR females. **Figure S8.** Top 15 highest ranked GO sets based on enrichment analysis of status-related DEGs in cerebellum of NMR females. **Figure S9.** Top 15 highest ranked GO sets based on enrichment analysis of status-related DEGs in testis of NMR males. Figure S10: Top 15 highest ranked GO sets based on enrichment analysis of status-related DEGs in skin of NMR males. **Figure S11.** Mitonuclear ratios in non-breeders and breeders per sex, tissue, and species. **Figure S12.** Top 15 highest ranked GO sets based on enrichment analysis of the non-redundant set of aging-related 20% quantiles that show the greatest interspecies difference in males (Tes, Skn). **Figure S13.** Top 15 highest ranked GO sets based on enrichment analysis of the non-redundant set of aging-related 20% quantiles that show the greatest interspecies difference in females (Hrt, Pit, Ovr). (PDF 1241 kb)

Additional file 2: **Table S1.** Age at death for NMRs and GPs. **Table S2.** Number of analyzed biological replicates per group. **Table S3.** Reason for exclusion of samples from further analysis. **Table S4.** Number of uniquely aligned RNA-seq reads. **Table S5.** Mean pairwise Pearson correlation coefficients between biological replicates in (A) naked mole-rat and (B) guinea pig. **Table S22.** Proportion of mitochondrial transcriptonal output. **Table S25.** Enrichment of age-related genes (Digital Ageing Atlas) in status-related DEGs. Only tissues having at least 50 DEGs were tested for enrichment. **Table S27.** Fisher’s exact test for overlap between DAA and upper quintile of status-related expression differences. **Table S10, S12, S15–17, S19–21, S28, S29.** Functional gene set enrichments analyses—see Overview tab for detailed description. **Table S7–9, S11, S13, S14, S18, S23, S24, S26.** Differentially expressed genes—see Overview tab for detailed description. (XLSX 313 kb)

Additional file 3: Text S1. Detailed description of expression changes in genes involved in steroidogenesis. Text S2. Detailed description of expression changes in gonadotropin-related genes (PDF 296 kb)

Additional file 4: Supplementary Data S1-S11 containing test results for differential expression between NMR vs. GP. (XLSX 5404 kb)

Additional file 5: Supplementary Data S12-S47 containing test results for differential expression between female vs. male in each species, group, and tissue (DESeq2). (XLSX 28655 kb)

Additional file 6: Supplementary Data S48-S87 containing test results for differential expression between breeder vs. non-breeder in each species, sex, and tissue (DESeq2). (XLSX 31326 kb)

Additional file 7: Gene annotation track for guinea pig. (GFF 19536 kb)

Additional file 8: Gene annotation track for naked mole-rat. (GFF 18958 kb)

## Abbreviations

DEG: Differentially expressed gene
ED: Expression differences
FDR: False discovery rate
FET: Fisher’s exact test
GO: Gene Ontology
GP: Guinea pig
GP-B-FvM: Comparison of GP breeder females vs. males
GP-F-BvN: Comparison of GP female breeders vs. non-breeders
GP-M-BvN: Comparison of GP male breeders vs. non-breeders
GP-N-FvM: Comparison of GP non-breeder females vs. males
NMR: Naked mole-rat
NMR-B-FvM: Comparison of NMR breeder females vs. males
NMR-BvN: Comparison of NMR breeders vs. non-breeders (both sexes)
NMR-F-BvN: Comparison of NMR female breeders vs. non-breeders
NMR-M-BvN: Comparison of NMR male breeders vs. non-breeders
NMR-N-FvM: Comparison of NMR non-breeder females vs. males
