# Supplementary material for: Naked mole-rat transcriptome signatures of socially suppressed sexual maturation and links of reproduction to aging
Source: BMC Biol. 2018 Aug 2;16:77. doi: 10.1186/s12915-018-0546-z (PMC6090939; doi:10.1186/s12915-018-0546-z)
Supplement: Supplementary file 1 — Figure S1. Identity of protein-coding sequences between human, mouse, GP, and NMR. Figure S2. Schema of collected tissues. Figure S3. Hierarchical clustering of gene expression profiles. Figure S4. Top 15 highest ranked GO sets based on enrichment analysis of cross-species DEGs between NMR and GP. Figure S5. Top 15 highest ranked GO sets based on enrichment analysis of sex-related DEGs that are shared between GP breeder and non-breeder. Figure S6. Top 15 highest ranked GO sets based on enrichment analysis of status-related DEGs in the ovary of NMR females. Figure S7. Top 15 highest ranked GO sets based on enrichment analysis of status-related DEGs in adrenal gland of NMR females. Figure S8. Top 15 highest ranked GO sets based on enrichment analysis of status-related DEGs in cerebellum of NMR females. Figure S9. Top 15 highest ranked GO sets based on enrichment analysis of status-related DEGs in testis of NMR males. Figure S10: Top 15 highest ranked GO sets based on enrichment analysis of status-related DEGs in skin of NMR males. Figure S11. Mitonuclear ratios in non-breeders and breeders per sex, tissue, and species. Figure S12. Top 15 highest ranked GO sets based on enrichment analysis of the non-redundant set of aging-related 20% quantiles that show the greatest interspecies difference in males (Tes, Skn). Figure S13. Top 15 highest ranked GO sets based on enrichment analysis of the non-redundant set of aging-related 20% quantiles that show the greatest interspecies difference in females (Hrt, Pit, Ovr). (PDF 1241 kb) [file 12915_2018_546_MOESM1_ESM.pdf]

Naked mole-rat transcriptome signatures of socially-suppressed sexual maturation and links of reproduction to ageing

Additional File 1, Supplement Figures

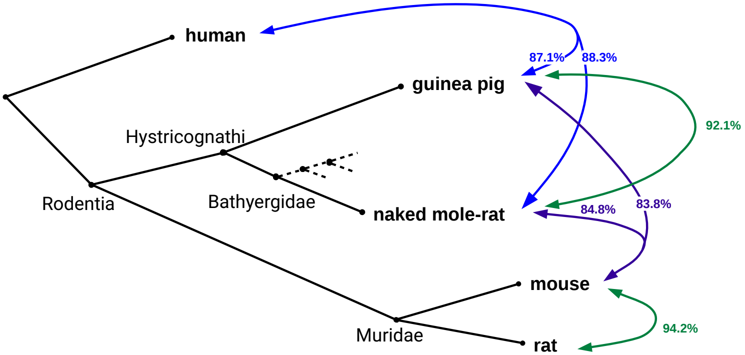

Supplement Figure S1. Identity of protein-coding sequences between different species. Guinea pig is much more similar to naked mole-rat than is mouse. Also, human shows higher sequence similarity to guinea pig and naked mole-rat than does mouse.

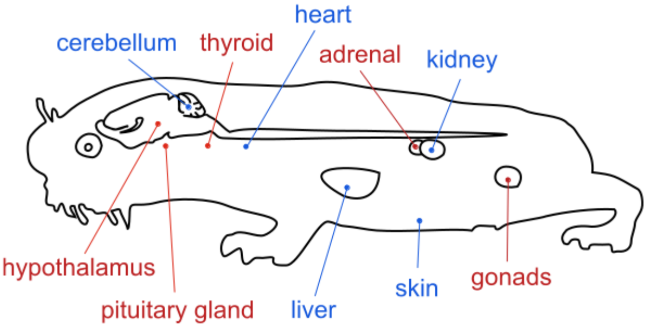

Supplement Figure S2. Collected tissues exemplified in schematic figure of NMR.

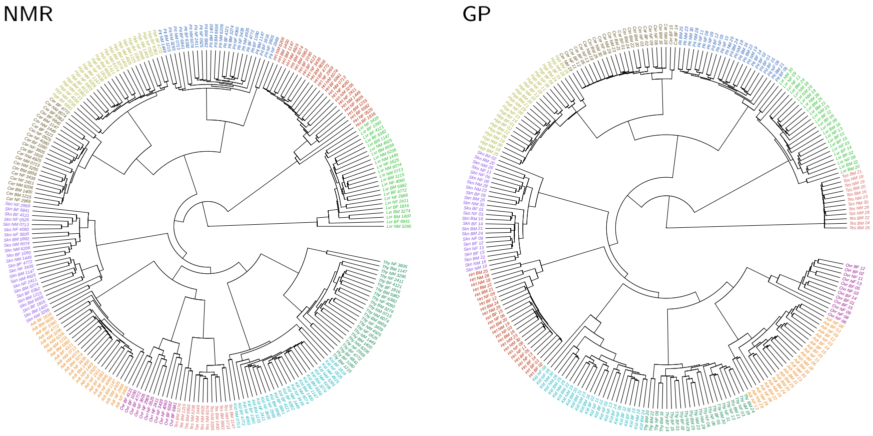

Supplement Figure S3. Hierarchical clustering of gene expression profiles. The clustering tree shows a clear separation of tissues in both species, but less pronounced differences between sex and breeding status.

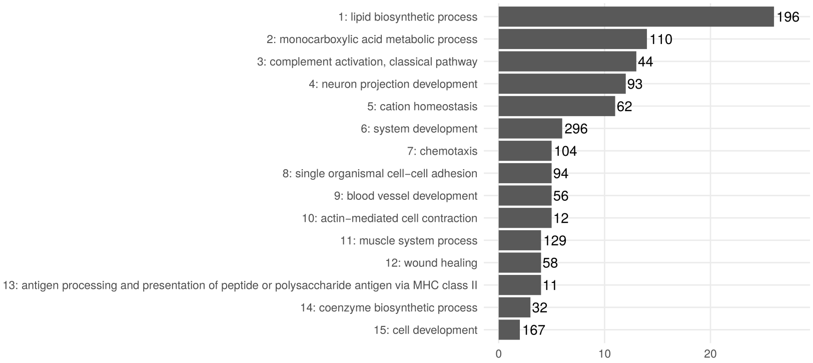

Supplement Figure S4. Top 15 highest ranked GO sets based on enrichment analysis of cross-species DEGs between NMR and GP. GO sets are ranked by number of summarized GO terms (x-axes) and number of DEGs (alongside bar).

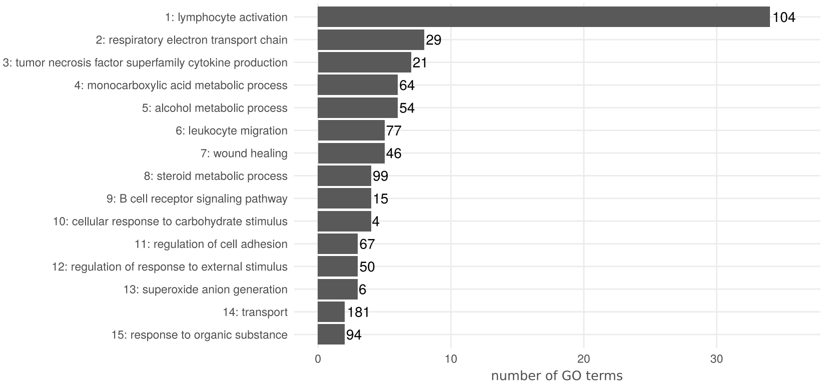

Supplement Figure S5. Top 15 highest ranked GO sets based on enrichment analysis of sex-related DEGs that are shared between GP breeder and non-breeder. GO sets are ranked by number of summarized GO terms (x-axes) and number of DEGs (alongside bar).

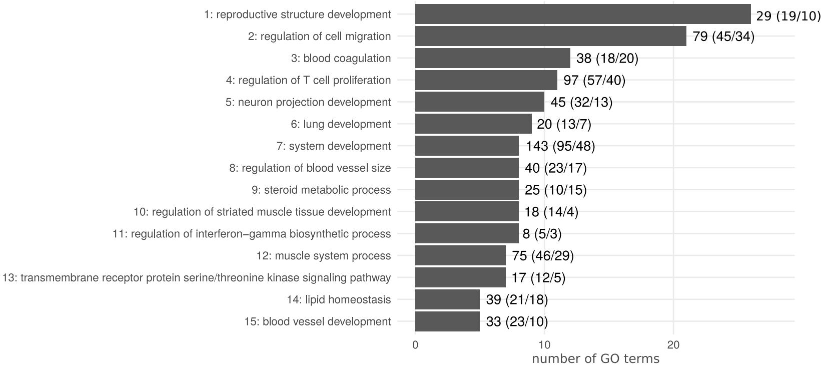

Supplement Figure S6. Top 15 highest ranked GO sets based on enrichment analysis of status-related DEGs in ovary of NMR females. GO sets are ranked by number of summarized GO terms (x-axes) and number of DEGs (alongside bar, together with number of up- and downregulated genes).

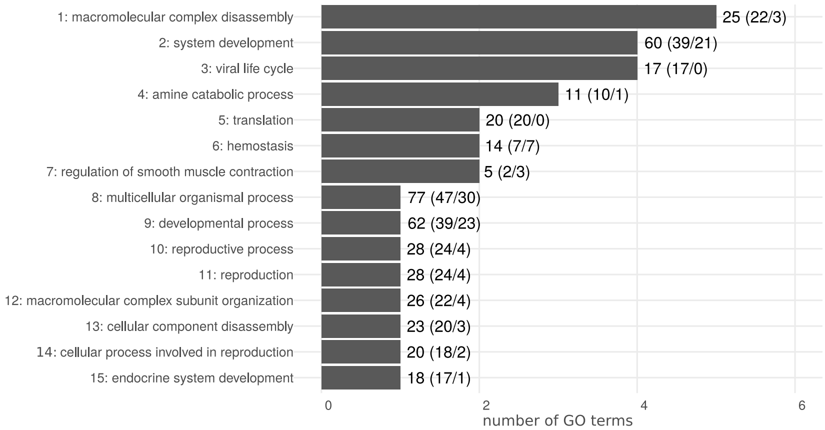

Supplement Figure S7. Top 15 highest ranked GO sets based on enrichment analysis of status-related DEGs in adrenal gland of NMR females. GO sets are ranked by number of summarized GO terms (x-axes) and number of DEGs (alongside bar, together with number of up- and downregulated genes).

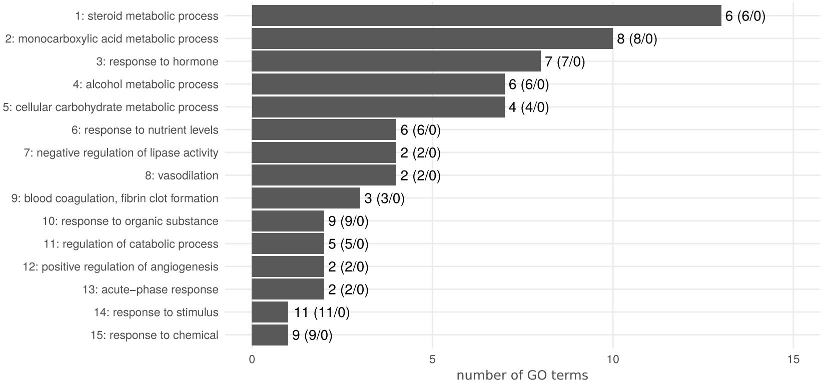

Supplement Figure S8. Top 15 highest ranked GO sets based on enrichment analysis of status-related DEGs in cerebellum of NMR females. GO sets are ranked by number of summarized GO terms (x-axes) and number of DEGs (alongside bar, together with number of up- and downregulated genes).

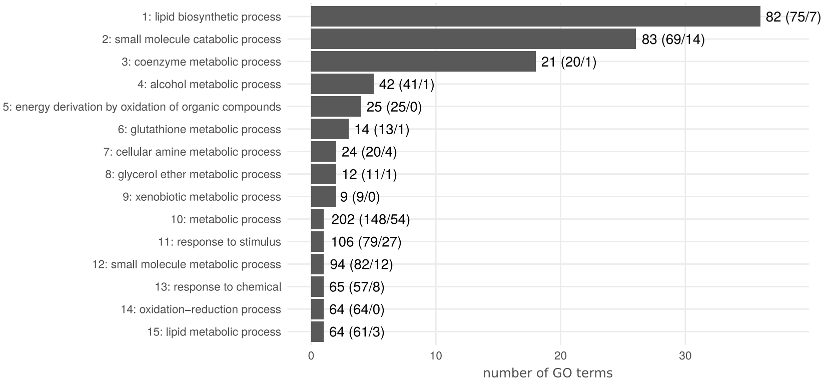

Supplement Figure S9. Top 15 highest ranked GO sets based on enrichment analysis of status-related DEGs in testis of NMR males. GO sets are ranked by number of summarized GO terms (x-axes) and number of DEGs (alongside bar, together with number of up- and downregulated genes).

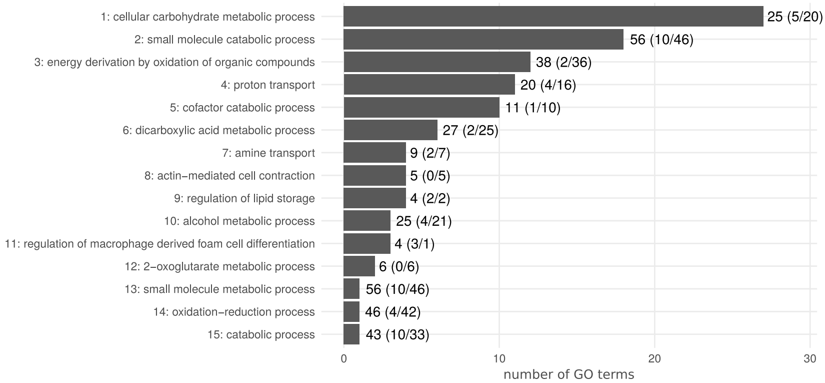

Supplement Figure S10. Top 15 highest ranked GO sets based on enrichment analysis of status-related DEGs in skin of NMR males. GO sets are ranked by number of summarized GO terms (x-axes) and number of DEGs (alongside bar, together with number of up- and downregulated genes).

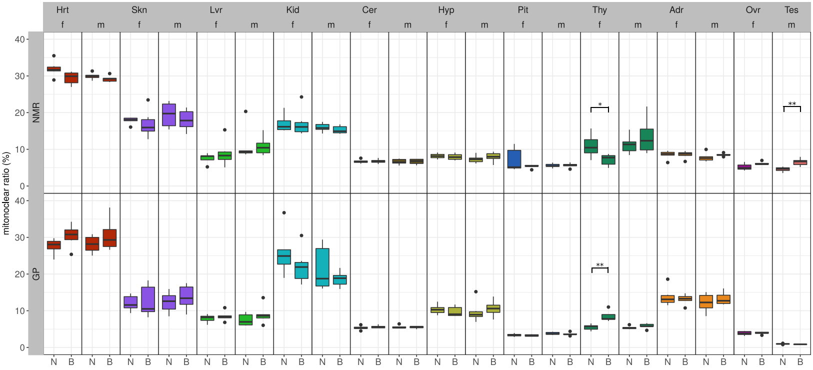

Supplement Figure S11. Mitonuclear ratios in non-breeders and breeders per sex, tissue and species. Boxplots shows median, 2nd/3rd quartiles, whiskers extend to 1.5 the interquartile range and dots values outside this range. P-values were calculated using a two-tailed t-test; *: p<0.05, **: p<0.01.

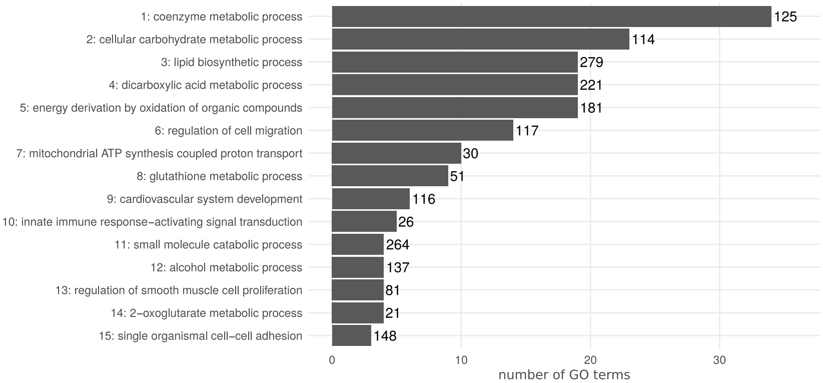

Supplement Figure S12. Top 15 highest ranked GO sets based on enrichment analysis of the non-redundant set of ageing-related 20%-quantiles that show the greatest interspecies difference in males (Tes, Skn). GO sets are ranked by number of summarized GO terms (x-axes) and number of DEGs (alongside bar).

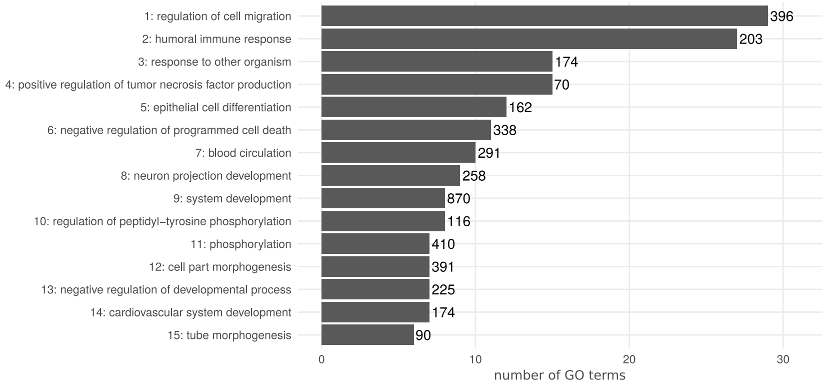

Supplement Figure S13. Top 15 highest ranked GO sets based on enrichment analysis of the non-redundant set of ageing-related 20%-quantiles that show the greatest interspecies difference in females (Hrt, Pit, Ovr). GO sets are ranked by number of summarized GO terms (x-axes) and number of DEGs (alongside bar).
