# Supplementary material for: Naked mole-rat transcriptome signatures of socially suppressed sexual maturation and links of reproduction to aging
Source: BMC Biol. 2018 Aug 2;16:77. doi: 10.1186/s12915-018-0546-z (PMC6090939; doi:10.1186/s12915-018-0546-z)
Supplement: Supplementary file 6 — Supplementary Data S48-S87 containing test results for differential expression between breeder vs. non-breeder in each species, sex, and tissue (DESeq2). (XLSX 31326 kb) [file 12915_2018_546_MOESM6_ESM.pdf]

Supplemental Text S1

In NMR-M-BvsN Tes, this is indicated by a significant upregulation of receptors for androgens (*AR*, 1.3-fold) and gonadotrophic hormones (*LHCGR*, 1.8-fold) as well as an increase in steroid production. Specifically, we observe an upregulation of genes involved in the transport of cholesterol into mitochondria (*STAR*, steroidogenic acute regulatory protein, 2.8-fold; *TSPO*, translocator protein, 1.5-fold; *CAV1*, caveolin 1, 1.4-fold), the rate limiting step of steroidogenesis (1), and conversion of cholesterol into pregnenolone (*CYP11A1*, cytochrome P450 family 11 subfamily A member 1, 1.8-fold), which is essential for all steroids. Genes encoding for enzymes that produce testosterone (*HSD17B1*, hydroxysteroid 17-beta dehydrogenase 1, 1.4-fold), the more potent metabolite dihydrotestosterone (*SRD5A1*, steroid 5 alpha-reductase 1, 1.3-fold) and cortisol (*CYP51A1*, cytochrome P450 family 51 subfamily A member 1, 2-fold) are upregulated as well (Supplement Figure 10). The upregulation of steroidogenesis is accompaanied by a significant increase in the mitonuclear ratio and enrichment of nuclear DEGs associated with mitochondria, including OXPHOS. Consequently, the load of reactive oxygen species (ROS) is presumably higher in NMR Tes, but is compensated by increase in antioxidant enzymes. NMR-F-BvsN Ova shows more subtle changes in steroidogenesis by increased estradiol production as indicated by significant upregulation of *CYP19A1* (aromatase, 2.5-fold) and increased sensitivity to androgens and progesterogens as indicated by upregulation of corresponding receptors (Ova, *AR*, 1,4-fold; Pit, *PGR*, 1.6-fold). We find, however, substantial expression changes in two genes involved in the regulation of follicle-stimulating hormone secretion (2), *INHA* (inhibin alpha subunit, 19.2-fold) and *INHBA* (inhibin beta A subunit, 4.4-fold), indicating that ovaries of non-breeding NMRs lack preovulatory follicles. Further, enrichment of steroid metabolic process, response to hormones and prostate gland growth indicates active feedback signalling also in females and an impact on developmental processes. Increase in signalling is further supported by significant enrichment of gene products located in secretory vesicle (secretory granule lumen, GO:0034774) and on cell surface (GO:0009986).

References

1. Stocco DM, Clark BJ (1996) Regulation of the acute production of steroids in steroidogenic cells. *Endocr Rev* 17(3):221–244.
2. Wijayarathna R, de Kretser DM (2016) Activins in reproductive biology and beyond. *Hum Reprod Update* 22(3):dmv058.

Supplemental Text S2

Although well expressed, we found no significant differences for gonadotropin-related genes (*GNRH1*, *FSHB*, *CGA*, *LHB)*. This indicates a similar intracellular transcript turnover in non-breeders and breeders, and is consistent with previous results indicating that LH is stored in non-breeders, ready to be released upon GnRH signalling (1). Notably, GnIH (NPVF), a negative regulator of reproductive function suggested to have a role in reproductive immaturity in NMRs due to its decreased protein expression in brain of NMR breeder (2), shows tendentially decreased mRNA expression in NMR breeders (males: -0.37, females -0.13). Also, previously elevated diencephalon mRNA levels in NMR were reported for *AR* (androgen receptor) in male breeders and for *CYP19A1* (aromatase), *ESR1* (estrogen receptor 1) and *PGR* (progesterone receptor) in female breeders (3). Our corresponding Hyp data do not support those increases (log2FC<0.1). Nevertheless, we identified significantly elevated transcript levels of these genes both in male (Tes: *AR*; Supplemental Data S67) and female breeders (Pit: *PGR*, Supplemental Data S54; Ova: *AR*, *CYP19A1*, *ESR1*, *PGR*, Supplemental Data S57) supporting a complex status-related function of these genes. Furthermore, steroid feedback and GnRH secretion are integrated by brain GABA and glutamate signalling in mammals and cichlids (4). Although, we do not observe equivalent significant differences in the NMR brain, the data show an upregulation of genes coding for three GABA receptor subunits (*GABRB3*, *GABRG1*, *GABRP*) and one glutamate receptor subunit (*GRIK2*) in Tes (Supplemental Data S67), as well as two of three differentially expressed GABA (*GABRB2*, *GABRG3*) and three glutamate receptor subunits (*GRIK1*, *GRIK2*, *GRIK4*) in Ova and female Pit of NMR breeders (Supplemental Data S54, S57). Together, this suggests increased neuronal plasticity and/or activity predominantly in gonads of NMRs after becoming breeders.

In the testis of immature chicken, expression of *ADIPOR1/2* (adiponectin receptor 1/2) is significantly lower than in mature animals (5). It has been hypothesised that these genes are involved in supporting the higher metabolic activity related to spermatogenesis, testicular steroid hormone production, and transport of spermatozoa and testicular fluid. In line with these observations, our results show a significant upregulation of *ADIPOR2* in Tes of NMR breeders (1.7-fold; Supplemental Data S67).

The renin-angiotensin system predominantly involved in cardiovascular control has also been associated with reproduction in mice and human (6). Signaling through receptors coded by *AGTR1* (angiotensin II receptor type 1) in males, and *AGTR1/2* in females has been associated with fertility and stimulation of reproduction. In accordance, we observed a significant upregulation of *AGTR1* and *AGTR2* in gonads of NMR male and female breeders, respectively (Supplemental Data S67,S57).

References

1. Faulkes CG, Abbott DH, Jarvis JU, Sherriff FE (1990) LH responses of female naked mole-rats, Heterocephalus glaber, to single and multiple doses of exogenous GnRH. *Reproduction* 89(1):317–323.
2. Peragine DE, et al. (2017) RFamide-related peptide-3 (RFRP-3) suppresses sexual maturation in a eusocial mammal. *Proc Natl Acad Sci* 3:201616913.
3. Swift-Gallant A, Mo K, Peragine DE, Monks DA, Holmes MM (2015) Removal of reproductive

suppression reveals latent sex differences in brain steroid hormone receptors in naked mole-rats, Heterocephalus glaber. *Biol Sex Differ* 6(1):31.

4. Renn SCP, Aubin-Horth N, Hofmann H a (2008) Fish and chips: functional genomics of social plasticity in an African cichlid fish. *J Exp Biol* 211(Pt 18):3041–3056.

5. Ocón-Grove OM, Krzysik-Walker SM, Maddineni SR, Hendricks GL, Ramachandran R (2008) Adiponectin and its receptors are expressed in the chicken testis: influence of sexual maturation on testicular ADIPOR1 and ADIPOR2 mRNA abundance. *Reproduction* 136(5):627–38.

6. Pan PP, Zhan QT, Le F, Zheng YM, Jin F (2013) Angiotensin-converting enzymes play a dominant role in fertility. *Int J Mol Sci* 14(10):21071–21086.
